# A Raman topography imaging method toward assisting surgical tumor resection

**DOI:** 10.1038/s44303-024-00006-6

**Published:** 2024-02-19

**Authors:** Alexander Czaja, Alice J. Jiang, Matt Zacchary Blanco, Olga E. Eremina, Cristina Zavaleta

**Affiliations:** 1 Department of Biomedical Engineering, University of Southern California, Los Angeles, CA, USA.; 2 Michelson Center for Convergent Bioscience, University of Southern California, Los Angeles, CA, USA.

## Abstract

Achieving complete tumor resection upon initial surgical intervention can lead to better patient outcomes by making adjuvant treatments more efficacious and reducing the strain of repeat surgeries. Complete tumor resection can be difficult to confirm intraoperatively. Methods like touch preparation (TP) have been inconsistent for detecting residual malignant cell populations, and fatty specimens like breast cancer lumpectomies are too fatty to process for rapid histology. We propose a novel workflow of immunostaining and topographic surface imaging of freshly excised tissue to ensure complete resection using highly sensitive and spectrally separable surface-enhanced Raman scattering nanoparticles (SERS NPs) as the targeted contrast agent. Biomarker-targeting SERS NPs are ideal contrast agents for this application because their sensitivity enables rapid detection, and their narrow bands enable extensive intra-pixel multiplexing. The adaptive focus capabilities of an advanced Raman instrument, combined with our rotational accessory device for exposing each surface of the stained specimen to the objective lens, enable topographic mapping of complete excised specimen surfaces. A USB-controlled accessory for a Raman microscope was designed and fabricated to enable programmatic and precise angular manipulation of specimens in concert with instrument stage motions during whole-surface imaging. Specimens are affixed to the accessory on an anti-slip, sterilizable rod, and the tissue surface exposed to the instrument is adjusted on demand using a programmed rotating stepper motor. We demonstrate this topographic imaging strategy on a variety of phantoms and preclinical tissue specimens. The results show detail and texture in specimen surface topography, orientation of findings and navigability across surfaces, and extensive SERS NP multiplexing and linear quantitation capabilities under this new Raman topography imaging method. We demonstrate successful surface mapping and recognition of all 26 of our distinct SERS NP types along with effective deconvolution and localization of randomly assigned NP mixtures. Increasing NP concentrations were also quantitatively assessed and showed a linear correlation with Raman signal with an R2 coefficient of determination of 0.97. Detailed surface renderings color-encoded by unmixed SERS NP abundances show a path forward for content-rich, interactive surgical margin assessment.

## INTRODUCTION

For the care of breast cancer patients, the first line of treatment is often surgical intervention. Of the approximately 200,000 newly diagnosed breast cancer patients each year in the United States, nearly 60% elect for a breast-conserving lumpectomy instead of mastectomy^1,2^. However, breast tumors suffer from poor tumor margin delineation, making them difficult for a surgeon to resect with complete negative tumor margins. Negative tumor resection margins, which are crucial for patient survival^3^, are achieved when a surgeon excises a tumor with an envelope of healthy tissue surrounding the malignancy, while positive margins occur when a surgeon cuts too close to or even through the cancerous lesion, leaving diseased tissue behind in the patient if not corrected. Positive tumor margins can occur in up to 60% of lumpectomy procedures and are associated with an increased rate of local recurrence^4-6^. There are currently no clinically adopted methods for intraoperatively detecting and correcting positive tumor resection margins for breast cancer lumpectomy in the operating room (OR). There are imaging guidance methods to help direct the surgeon to the tumor site, including preoperative magnetic resonance (MR), preoperative and intraoperative ultrasound, and mammography to navigate by calcifications and radiological findings. Faxitron imaging is used to confirm removal of a clip placed during biopsy, but positive margins still occur despite these facilities^7-10^. Touch preparation (TP) cytology has not provided reliable results for evaluating breast cancer resection margins, and the adipose content of breast tissue prevents it from being cryopreserved for rapid histopathology^11-15^. This means positive margins are only discovered postoperatively by a pathologist performing traditional formalin-fixed paraffin-embedded (FFPE) histopathology. The pathologist evaluates tissue morphology and biomarker expression stains under a microscope to discover whether any signs of malignancy exist at the specimen’s perimeter. Since pathologists cannot reasonably assess every thin section of a large specimen, it is estimated that less than 1% of a lumpectomy’s perimeter is assessed for the presence of positive margins^16-20^. There is a definite need for a tool which can interrogate the entire surface of a freshly excised lumpectomy specimen to reveal positive margins during initial surgical intervention and guide the surgeon to the remaining malignant tissue in the operation site.

Imaging surgical guidance to assist in the location and resection of solid tumors is a promising and growing field of research with direct implications to patient outcomes^21-26^. Advancements in tailored intraoperative imaging approaches using knowledge of cancer pathophysiology are helping to direct surgeons to the disease and achieve more precise resections. Breast cancer is a good candidate for molecular imaging approaches as it often presents with a different protein expression profile than healthy tissue^27-31^. Multiplexed optical imaging methods involve staining a specimen with distinguishable contrast agents which bind to abnormal or overexpressed proteins, washing away any unbound material, and placing the specimen in an imaging apparatus to visualize any foci of abnormal biomarker protein expression. Previously, we have shown that Raman spectral imaging is a highly capable modality for multiplexed molecular imaging applications thanks to the contrast agent recognition specificity afforded by the distinct nature of every molecular species’ vibrational characteristics^32-38^. Raman spectroscopy entails measuring light which has been Stokes-shifted relative to the incident light due to the promotion of a sample’s molecules to virtual energy states and relaxation to quantum vibrational states which are characteristic of their composition of atoms, bonds, and symmetries thereof. It only requires the use of one incident wavelength, while optical methods like fluorescence imaging tend to require many excitation wavelengths and emission filters to achieve multiplexing. The surface-enhanced Raman scattering (SERS) phenomenon increases the inelastic scattering cross section of molecules in the vicinity of nanotextured metal substrates by many orders of magnitude, meaning nanoparticle (NP) contrast agents can be fabricated carrying a bundle of molecules of some species with desirable spectral properties^39-41^. SERS NPs can be made to produce different spectral emissions by swapping the reporter molecule species introduced between the metallic core and the protective silica shell.

It has been shown that gold-core SERS NPs can be functionalized with cancer biomarker-targeting antibodies^34,42-45^. The SERS NP fabrication process is summarized in Fig. S1. Batches of SERS NPs encapsulating different reporter molecules can be respectively functionalized with different antibodies, and these batches can then be mixed into a cocktail of SERS NP labels which each preferentially bind their complementary biomarker protein antigens. With reference spectra measured from the separate pure SERS NP batches, quantitative spectral unmixing postprocessing can determine how much of each SERS NP label bound to the stained surface. An additional SERS NP batch can be included in the staining cocktail functionalized with an isotype control antibody to provide a nonspecific binding control channel. Biomarker expression levels can then be quantitatively determined using the ratio of bound biomarker-specific SERS NPs to the amount of isotype control SERS NPs in each pixel^43-45^. Cancer biomarker proteins interrogated in diagnostic staining and molecular imaging are often ones whose overexpression enables cancer growth mechanisms like rapid cell division, prevents apoptosis, or encourages angiogenesis^46^. Abnormally high expression levels of these proteins can signify where cancerous cells reside and which proteins and pathways they may be exploiting to rapidly divide.

In fact, Wang et al. demonstrated that a 5-plex cocktail of antibody-functionalized SERS NPs could quantify the presence and distribution of overexpressed biomarker proteins from tissue shavings excised from breast cancer patients^43-45^. Biomarker protein expression levels and spatial distributions were validated with gold standard immunohistochemistry (IHC). Across those studies, the 5 interrogated biomarkers included epidermal growth factor receptor (EGFR), human epidermal growth factor receptor 2 (HER2), estrogen receptor (ER), CD24, and CD44. The staining cocktails stained for 4 biomarkers at most and included an isotype control SERS NP type for ratiometric quantitation purposes. Wang et al. generated receiver operating characteristic (ROC) curves comparing ratiometric SERS NP quantitation of biomarker expression against gold standard IHC. The combined detection of these biomarkers allowed Raman imaging to detect breast carcinoma with a sensitivity of 89.3% and specificity of 92.1%^45^. These results, highlight the potential of this multiplexed targeted strategy to guide surgical resection and thus motivated our approach to offer whole specimen surface mapping of the entire resected tissue. It is important to note that the fresh tissue specimens stained and imaged in these previous studies were flat tissue shavings sampled from the patient’s surgical cavity after the surgeon had removed the main mass, making it tedious to interrogate positive tumor margins throughout the entire surgical cavity.

**Fig. 1:**
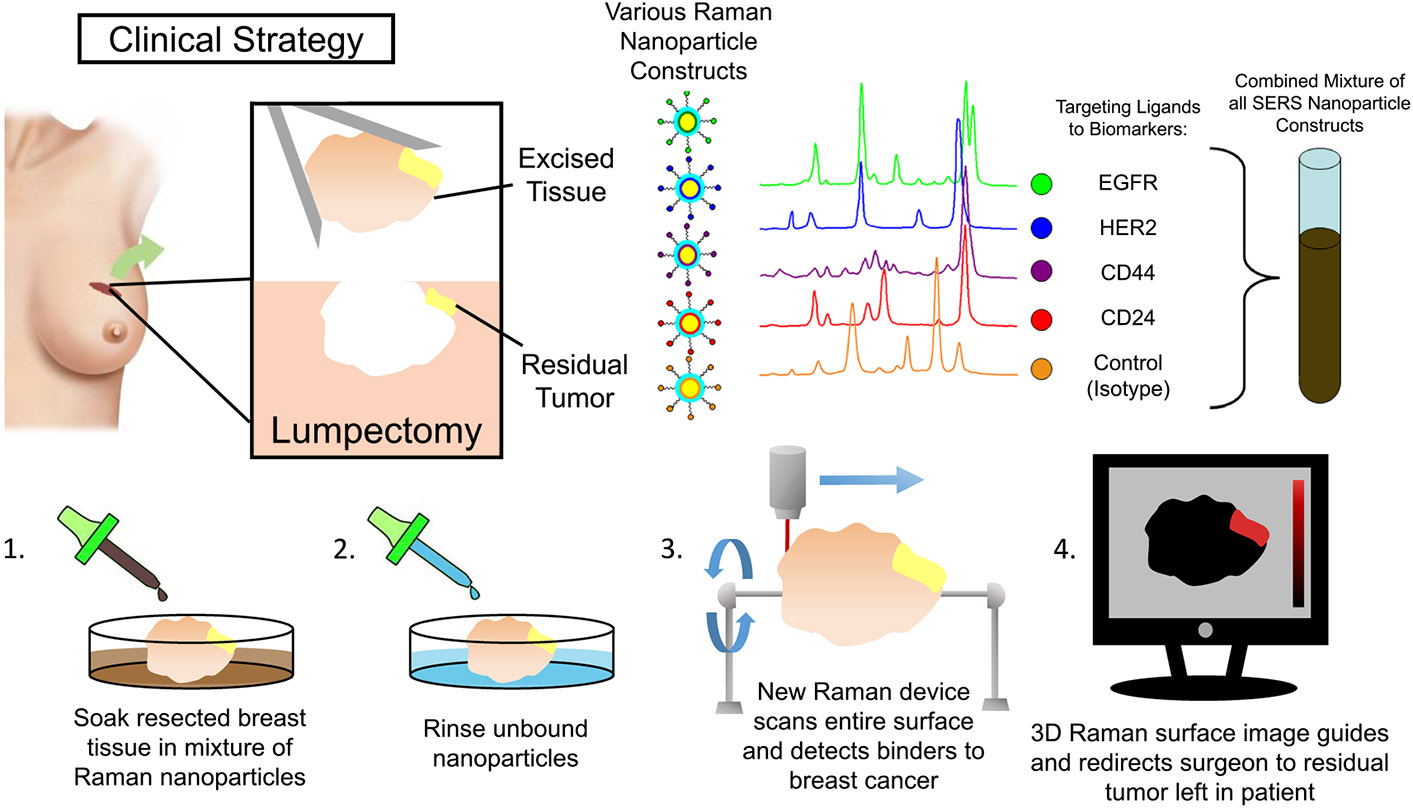
Proposed surgical workflow. If a breast cancer lumpectomy is resected with unfavorable positive margins, traces of malignant tissue are likely on the specimen’s surface. Detecting and locating its presence while the patient is still in the OR could rapidly inform the surgical team where additional tissue should be removed to achieve a more complete excision. SERS NPs consisting of different reporter molecules and chemically conjugated with biomarker protein-targeting moieties can serve as multiplexed spectral imaging contrast agents. A freshly excised specimen could be incubated with a cocktail of such NPs, rinsed with fresh buffer to wash away unbound material, and topographically imaged. The resulting spectral and spatial information could be rendered in such a way to inform a surgical team whether any positive margins exist and where to remove additional tissue from the surgical cavity.

We propose an intraoperative molecular imaging approach which directly interrogates the entire surface of a freshly excised breast cancer lumpectomy specimen. Our method allows us to measure full Raman spectra concurrently with coordinates of surface points, allowing us to generate a 3D topographic image of a specimen’s size, shape, texture, and SERS NP content. Readily available Software packages can rotate, zoom, and pan 3D object renderings, so a surgical team could easily manipulate and inspect the entirety of a Raman topography image of a lumpectomy specimen to locate regions of abnormal protein expression and direct removal of additional tissue. Imaging the fresh surface of a lumpectomy specimen circumvents the problem that breast tissue is incompatible with the method of rapid biomarker interrogation currently practiced in the clinic. This approach would directly interrogate the original resection margin the surgeon made while balancing the need to remove the tumor while conserving surrounding tissue. It would also avoid the need to remove tissue shavings from the resection site without guidance as to whether or where shavings would be beneficial to the patient. There are commercial Raman imaging systems which comprise focusing lasers and high-resolution stages with adaptive autofocusing capabilities to adjust the stage’s z-coordinate automatically and independently as the stage moves in the xy-plane to maintain or regain focus on the sample’s surface when subsequent pixel locations are reached. We have developed an accessory tool for such a Raman imaging system which incrementally rotates a specimen in synchrony with lateral stage movements and spectral acquisitions to expose all surfaces of the specimen for interrogation. Figure 1 illustrates this proposed surgical workflow for intraoperative tumor resection margin imaging. Such an approach would allow for interrogation of the resected lumpectomy specimen and provide information of the surface’s cellular makeup with all the spatial context necessary to make an informed decision of where to continue excision within the surgical cavity if complete tumor resection was not achieved.

Here, we present the conjunction of our newly developed rotational accessory device and a commercial Raman imaging system which enables complete topographic surface imaging. 3D rendering fidelity is validated against the known shape and texture of phantom objects prepared from moldable modeling material, and we demonstrate the ability of distinct SERS NP types to be located and identified to correlate a sample to its excision orientation. Multiplexed topographic images validate identification specificity of many distinct SERS NP types when separated and when mixed in the same locations. Finally, we validate that topographic imaging is achievable using fatty porcine tissue as an analog of adipose-rich human breast tissue and confirm that SERS NPs can be both qualitatively and quantitatively detected against biological tissue background signals.

## METHODS

### Raman microscope and topographic imaging accessory

A commercially available Renishaw RA816 Biological Analyser was used for all Raman spectral imaging. All measurements were performed with a Leica N PLAN L 50 × 0.50 NA objective lens with a working distance of 8.2 mm, a Renishaw MS 20 encoded stage, and a 785 nm diode laser set to a power of 10 mW. Renishaw’s autofocus system, which continuously adjusts the stage’s z-position to maintain adequate focus on a sample’s surface, was utilized for all topographic imaging. With this, every pixel’s spectrum is only acquired once autofocus adjustments have converged. The Raman imaging system is controlled by Renishaw’s Windows Raman Environment (WiRE) software package which allows users to define pixel step sizes, laser power, and exposure time parameters when executing Raman images. WiRE also implements postprocessing functions such as correction of spectral baseline and quantitative least-squares regression spectral unmixing for spatial chemometric analysis. Raman spectral image records contain the list of Stokes Raman shifts at which each spectrum in the image was measured in units of wavenumber relative to the incident light wavenumber, the scattered light intensities of each spectrum in arbitrary units, and every pixel’s the x-, y-, and z-dimension stage coordinates.

WiRE presents an external control interface which allows other entities to interact with it via a standard networking protocol (TCP/IP). External control can be used for tasks like enqueuing and dequeuing measurements, monitoring the status of running measurements, or triggering the spectral acquisitions of a particular Raman image’s pixels based on input from additional code or auxiliary input sources. With triggering enabled, the Raman imaging system moves the stage in the xy-plane to the next pixel’s location, makes any z-position adjustments to maintain focus on the sample’s surface, and enters a waiting state until triggered. The waiting state can be queried by another program or system connected to the WiRE server. With this facility, we implemented a script which sends serial USB commands to our accessory to perform angular steps and triggers WiRE to collect individual spectra each time WiRE enters a waiting state. Each pixel’s spectrum acquisition is contingent on both an external trigger signal and the autofocus system having completed any z-dimension stage adjustments to maintain focus on the surface. The autofocus system engages to ensure focus after lateral stage movements and angular sample movements. WiRE concludes and dequeues the image once all pixels’ spectra have been collected.

To acquire a Raman topography image, the sample is placed on the accessory device rod, the user defines beginning and ending x-coordinates for the image, sets the desired lateral and angular step sizes, and executes the external control script. The script manages enqueuing the image, monitoring for WiRE entering a waiting state, executing angular steps, and triggering spectral acquisitions. A sample is stepped in the x-dimension, rotated about its x-axis, and adjusted to maintain focus in the z-dimension between each spectrum acquisition during topographic imaging. Spectra are measured by the downward-facing objective lens in a spiraling, corkscrew fashion. There is no need in this scheme for any y-dimension stage movement. A cylindrical coordinate system conveniently represents each acquisition’s location on a specimen’s surface. The z-coordinates in the Raman image record represent the radial dimension of the cylindrical coordinate system because we set the Raman imaging system’s origin at the center of the accessory rod. The x-coordinates represent the cylindrical coordinate system’s axial dimension, and the angular dimension is known by the user’s angular step setting.

**Fig. 2:**
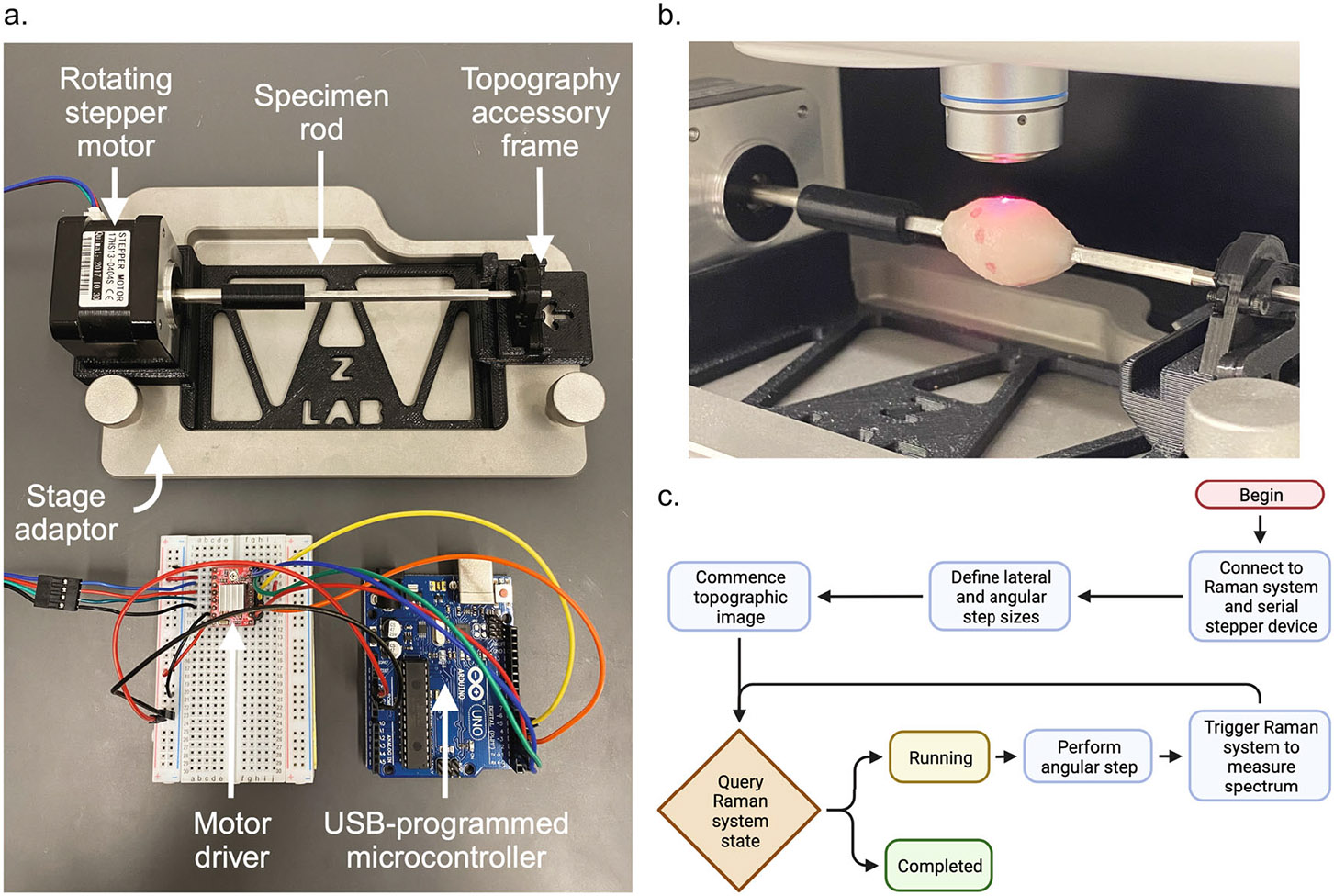
Topographic imaging device. 3D topographic Raman imaging is achieved by the combination of a device which iteratively rotates a sample in synchrony with a commercial Raman imaging system. **a** Annotated photo of the topographic Raman imaging accessory. **b** Image of the Renishaw RA816, its objective lens, and its laser focused onto the surface of a tissue specimen coated with SERS NPs on the accessory. **c** Diagram of external control script loop which synchronizes the angular movements of the accessory with the lateral stage movements and spectral acquisition of the Raman imaging system.

Figure 2 illustrates a device where samples can be affixed on a rod suspended between the microscope stage and objective lens. Figure S2 shows a wider view of the Raman topographic imaging accessory situated in the Renishaw RA816 Biological Analyser. The rod has a square cross-section profile to prevent sample slipping during imaging and is made of stainless steel for easy cleaning and sterilization. The device frame, 3D printed with polylactic acid (PLA), is situated inside a stage adapter holding the rod above the floor of the stage adapter while maintaining a low enough profile to allow sufficient z-dimension travel for topographic imaging of large specimens. The height of the device, z-dimension travel of the stage, and the objective lens working distance afford enough vertical space to accommodate topographic imaging of specimens in the size domain of real breast cancer lumpectomy specimens^9,10^. The frame secures an angular stepper motor (Nema 17, 1.8° native step) and supports the free end of the stainless-steel rod. A consumer microcontroller (Arduino Uno) is programmed, powered, and controlled by a standard USB connection to the workstation computer and supplies power to the stepper motor. The microcontroller is wired to the pins of a motor driver chip (A4988, Allegro MicroSystems) which modulates the power supplied to the motor, governs the direction and size of angular steps by setting the motor’s pins, and provides pins to fractionally divide the motor’s native step size to optionally increase angular resolution.

With this spiral topographic imaging scheme, the spacing between discrete pixel acquisitions can be controlled by setting the angular step size of the accessory and the stage’s x-dimension step size. The angular spacing will govern how many samples occur per revolution. Samples’ angular spacing is an arclength function of the sample’s local radius and the step angle. The x-dimension spacing governs the lateral spacing between individual pixels as well as the spiral’s pitch, or axial spacing. The spiral’s pitch is the product of the x-dimension step and the number of angular steps per full revolution. Spiral acquisition parameters stepping between each pixel were set to a lateral step of 1 μm and an angular step of 0.9° for all topographic images. Supplemental Video 1 shows our Raman topography system as it acquires images with automatic autofocus adjustments during acquisition.

### Topography phantoms

To develop and validate this whole-surface topographic approach to Raman imaging, we adopted simple polymer modeling material as a moldable imaging phantom substrate. It can easily be prepared according to any desired size and surface texture specifications, be coated with SERS NPs, and be stored for long periods without degradation. Topographic imaging phantoms were made with this modeling polymer stamped with various patterns to validate the fidelity and surgical workflow utility of the resulting 3D surface renderings. We also coated these phantoms with sets of SERS NPs to validate their localization and multiplexing specificity. Finally, we validated our ability to perform topographic Raman imaging and to localize and quantitate SERS NPs with organic tissue. We used commercially available porcine tissue as an organic phantom analog for breast tissue for its mixture of rich adipose and muscle tissues. Phantoms were approximately 2 cm in diameter.

### 3D rendering

All spectra were quantitatively unmixed with nonnegatively-constrained least-squares (NNLS) regression. Unmixing results are coefficients, or relative weights, by which reference spectra are multiplied and summed to best approximate an image’s multiplexed spectra. These unmixed coefficients make up what we interpret as an image channel depicting the amount and spatial distribution of a substance. Though these topographic datasets are collected as images of a single row as far as the WiRE system is concerned, we can use the knowledge of how many samples make up a full spiral period to produce 2D images by populating a series of columns. Unmixed channels can be assigned pseudocolors to distinguish the presence of different SERS NPs. Point clouds of each pixel’s coordinates with corresponding quantitatively attenuated color values were written to Stanford PLY files^47^. Coordinates in units of microns were converted from the original cylindrical system to a Cartesian system. Point clouds were visualized with the MeshLab software package using ball-pivoting surface reconstruction, and Taubin smoothing^48-50^. This process takes the point cloud to be vertices of a graph and determines a set of triangles whose edges and faces connect the vertices to render a surface. Triangle faces were colored according to the colors of the vertices they connect.

### SERS NP fabrication

The SERS NPs comprise a gold nanoparticle core (AuNP), a Raman reporter layer, and a protective silica shell. Briefly, bare AuNPs were fabricated by a hydroxylamine seeding method. 3 ml of 30 mg/ml gold(III) chloride was added with vigorous stirring to 450 ml of 4 °C water followed by rapid addition of 0.6 ml of a 0.135 mg.ml sodium citrate and 0.085 g/ml hydroxylamine hydrochloride solution. 10 s later, 0.12 ml of a 0.001% NaBH_4_ solution was rapidly added. After stirring for 10 min, 25 ml of 37.5 pM AuNPs were diluted with an equal volume of water 40 μl of 1 mM (3-aminopropyl)trimethoxysilane (APTMS) was added dropwise. 15 min of stirring later, an ethanol solution of a Raman reporter species was added to form the Raman-active layer. SERS labeling was confirmed with the Raman spectral imaging system. After 5 min of stirring, 500 μl of 2.15 wt% sodium silicate was added, stirred for 15 min, and allowed to rest for 24 h. 30 nm silica shells were grown with the Stöber method by addition of 125 ml ethanol, 625 μl ammonia added dropwise, and 50 μl of tetraethyl orthosilicate (TEOS) and allowing stir for 24 h at room temperature^51^. Thiolation of SERS NPs was achieved by an additional Stöber process with (3-mercaptopropyl)trimethoxysilane (MPTMS). SERS NPs were purified and concentration by 5 × centrifugation at 1500 g for 30 min. A maximum absorbance band of the AuNP colloidal solution at 535 nm was confirmed by UV-vis spectrophotometry (Cary 60, Agilent Technologies, UCA). Dynamic light scattering (DLS) (Zeta Sizer Nano ZS, Malvern Panalytical, UK) and nanoparticle tracking analysis (NTA) (NanoSight NS300, Malvern Panalytical, UK) were used to measure AuNP concentrations and to characterize their size distribution to be 61 ± 4 nm. Silica-coating of SERS NPs was characterized by transmission electron microscopy at 80 kV, 15,000 × magnification (TEM; JEOL 1200ex-II, Japan).

### Chemicals and NPs

Reagents Gold(III) chloride hydrate (HAuCl_4_·xH_2_O, 99.995%), trisodium citrate dihydrate (C_6_H_5_Na_3_O_7_·2H_2_O, 99.0%), (3-amino-propyl)trimethoxysilane (APTMS, 98%), 5-amino-1,3,4-thiadiazole-2-thiol (ATDT, 98%), 1,2-bis(4-pyridyl)ethylene (BPE, 97%), 4,4′-bis(mercaptomethyl)biphenyl (BMMBP, 97%), 4-(trifluoromethyl) thiophenol (TFMTP, 99%), 4,4′-dipyridyl (44DP, 98%), d8-4,4′-dipyridyl (d8-44DP), 4,4′-azopyridine (44AP, 99%), 1,2-bis(4-pyridyl)acetylene (BPA), phthalazine (PHTH, 98%), 4-mercaptopyridine (4MPY, 96%), 2-mercaptobenzothiazole (2MBT, 97%), 4,4′-thiobis-benzenethiol (TBBT, 98%), 4-aminothiophenol (ATP, 96%), 4-nitrothiophenol (NTP, 99%), 2-bromothiophenol (BTP, 97%), benzyl mercaptan (BMP, 99%), 6-amino-2-mercaptobenzothiazole (AMBT, 97%), 2,5-bis(4-pyridyl)-1,3,4-thiadiazole (BPT, 97%), sodium silicate aqueous solution (~26.5%), and tetraethyl orthosilicate (TEOS, 99%) were purchased from Sigma-Aldrich. Reagent 5,5′-dithiobis(2-nitrobenzoic acid) (DTNB, 99%) was purchased from Beantown Chemical. Reagent 5-(4-pyridyl)-1,3,4-oxadiazole-2-thiol (PODT, 97%) was purchased from Alfa Aesar. Reagents 5-(4-pyridyl)-1H-1,2,4-triazole-3-thiol (PTT, 98%), thiophenol (PhSH, 99%), 2,2′-dipyridyl (22DP, 99%), 2-naphthalenethiol (2NT, 99%), and 4-mercaptobenzoic acid (4MBA, 90%) were purchased from Acros Organics. All work used deionized water (Milli-Q grade, Millipore) with a resistivity of 18.2 MΩ cm.

## RESULTS

### Topographic image validation

**Fig. 3:**
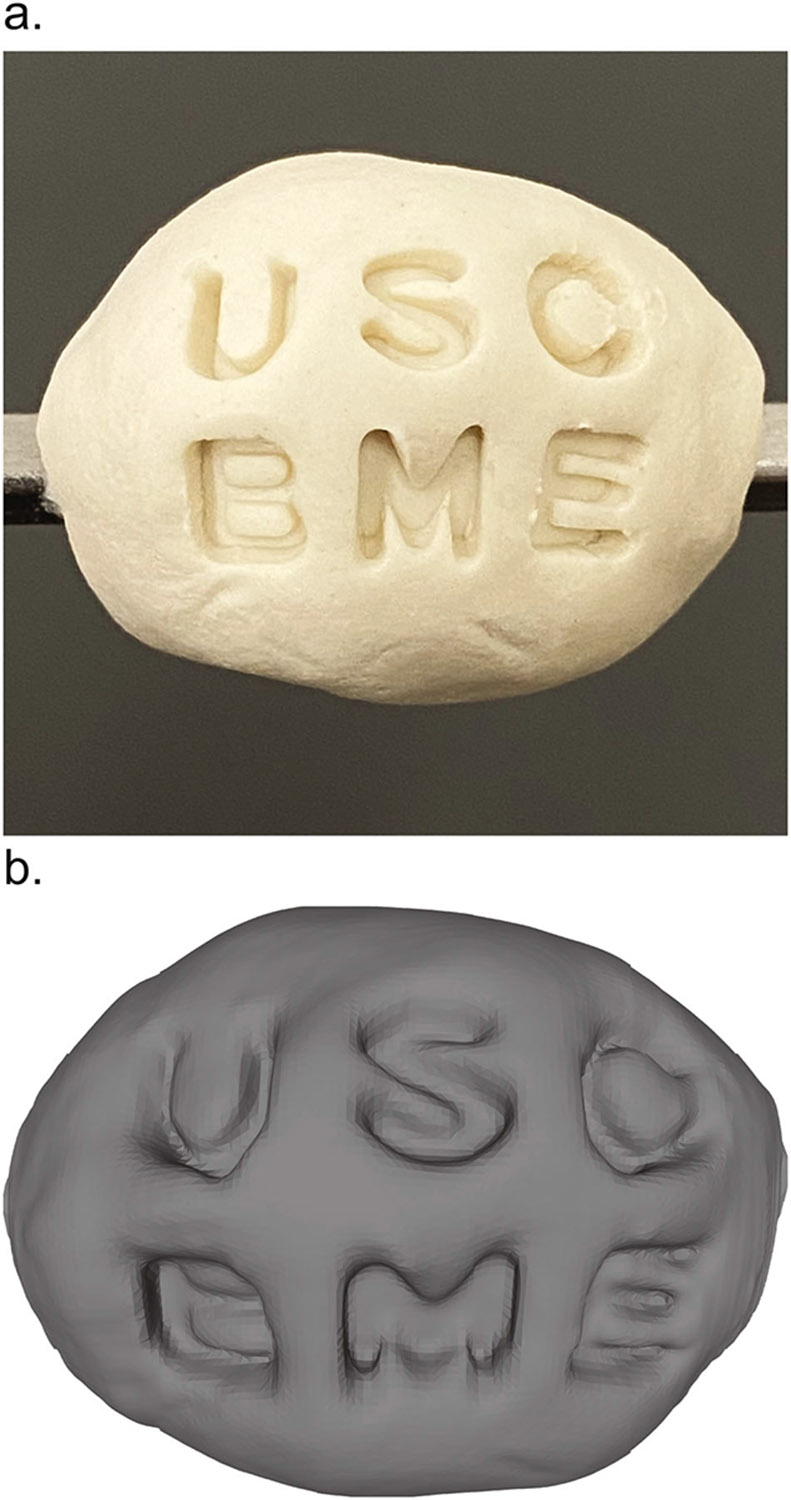
Topography imaging validation phantom. An object’s topography is captured and rendered by the spiral acquisition protocol and point cloud surface mesh reconstruction, respectively. **a** A phantom object molded from polymer modeling material and stamped with a test pattern. **b** Resulting topographic image 3D rendering of the phantom’s surface.

To begin validating that the visual characteristics of a sample, such as its perceived size, shape, and surface texture, could be adequately captured by our topographic imaging approach, we created and imaged a simple clay phantom impressed with an identifiable test pattern (Fig. 3). The letters “USC BME” are prominently stamped into its surface, and this texture is clearly recognizable in the resulting 3D surface rendering (Fig. 3b). Replicating the shape and structural landmarks of sample with this topographic surface sampling method in a 3D rendering is a key component of orienting, navigating, and interpreting the resulting image. During topographic imaging, when the valleys of the stamped test pattern are encountered by the Raman imaging system, the autofocus system notices the stage should be moved in the positive z-direction to compensate for the dip in the specimen’s surface to achieve optimal and consistent collection of Raman-scattered light. Conversely, once the image has progressed where the laser exits a feature of the impressed test pattern, the autofocus system adjusts the stage in the negative z-direction.

### Specimen orientation validation

**Fig. 4:**
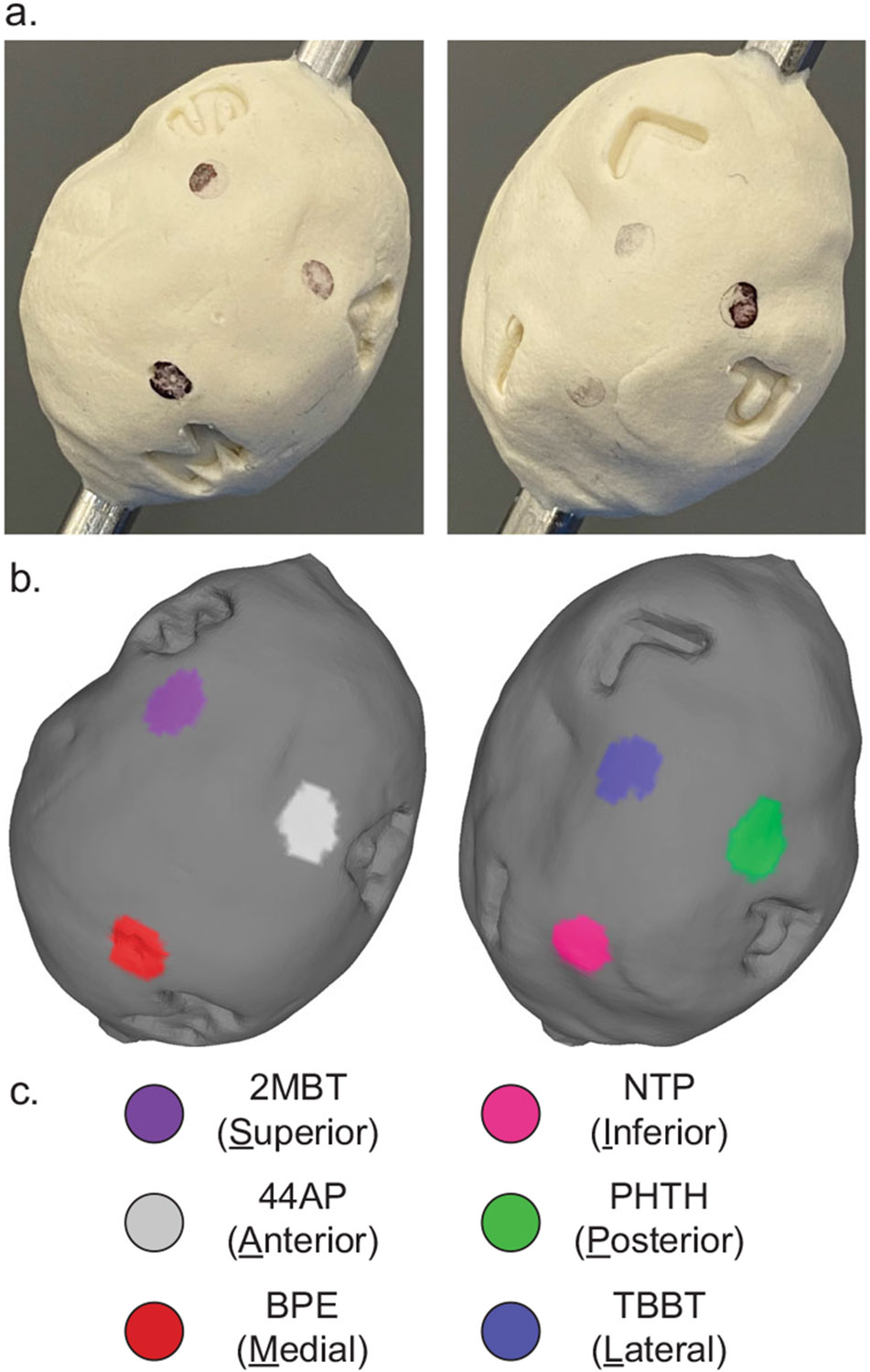
Anatomical orientation validation. Topography and SERS NP contrast agents can be visualized simultaneously. SERS NP contrast agents can be used to orient a specimen’s image as expected by standard of care practices. **a** Lettered stamps indicating hypothetical superior, inferior, anterior, posterior, medial, and lateral faces of a specimen. The two views of the specimen are rotated about the specimen’s x-axis by 180°. **b** Color-coded topographic image rendering clearly depicting distinct SERS NP identities located in the vicinity of respective surface impressions. **c** Legend correlating color to a Raman reporter species.

Resected tumor specimens are typically “inked” to annotate their surfaces with which surgical site its faces were in contact with as part of the standard of care tissue processing workflow^10,52^. This practice orients it to the patient’s and resection site’s anatomy, allowing the care team to trace definitively any positive margins discovered in postoperative histopathology back to a particular site and direction where the revision surgery should focus on removing additional tissue. Here, we demonstrate topographic imaging of another molded validation phantom stamped with letters signifying the six standard anatomical positions marking a hypothetical specimen orientation (Fig. 4). Each face also received a coating of a different SERS NP type each bearing a distinct Raman spectrum. The size, shape, surface texture, and position of recognizing the presence of distinct SERS NP types confirms that Raman imaging contrast agent materials can be located with specificity and precision with topographic imaging. A video clip of our newly developed rotational accessory tool is depicted in Supplemental Video 1 as it acquires a topographic Raman image with active autofocusing.

Note the ease of use to manipulate and inspect a 3D rendered Raman topography image volume in Supplemental Video 2. A user can easily control and interact with the resulting 3D rendering at a viewing workstation. The indentations and shadows of the rendering depict the actual appearance of the object and how the faces and features of a tissue specimen would appear. An entire rendered surface can be rotated and zoomed for a detailed inspection. The SERS annotation markings on a tumor could easily orient the user to its original position within the surgical cavity. Any locations of increased biomarker protein binding, which would appear as a surface region of increased intensity of another color, can be fully described by the nearest anatomical SERS annotations. These coordinates are intended to reorient the surgeon to the corresponding areas in the surgical cavity and precisely direct them to where further resection is needed to achieve complete removal of the tumor.

An intraoperative imaging approach must consider the expectations of OR and pathology teams to maintain patient safety, tissue integrity, and compatibility with standard of care tissue processing and pathology. Staining of fresh tissue with antibody-conjugated SERS NP contrast agents has been shown to require as little as 15 min of incubation^44^. These Raman topography images are able to be collected and visualized in less than an hour, which is sufficiently quick for the patient to remain anesthetized without significantly increased risk and for tissue specimen integrity not to degrade before undergoing standard pathology processing. The standard of care approach for resected tumor processing is for it to be inked with different colors indicating the original anatomical orientation of each face^53^. A surgeon may also mark the faces of a specimen first with distinct sutures prior to inking. If pathology discovers malignant cells sufficiently close to the specimen perimeter, the perimeter color indicates the direction in which the surgeon should remove additional tissue in a later surgery. SERS NP staining and topographic imaging of biomarker protein expression could take place before inking or after by placing at the suture locations additional untargeted SERS NPs carrying Raman reporters not used for functional labeling after incubation in the staining cocktail. Alternatively, if inking is done before Raman topography, the Raman spectra of the inking dyes themselves may even provide sufficient signal themselves for orientation. When color-coded topographic imaging results are being interpreted by the team, they can also be presented with a color-coded rendering showing the locations of the orienting contrast agents. Any foci of abnormal targeted biomarker binding can then be definitively located by their nearest orienting signals to immediately inform where additional tissue should be excised in the surgical cavity. The specimen could then be removed from the topography device rod and sent for traditional pathological assessment.

### Availability of large SERS NP library

**Fig. 5:**
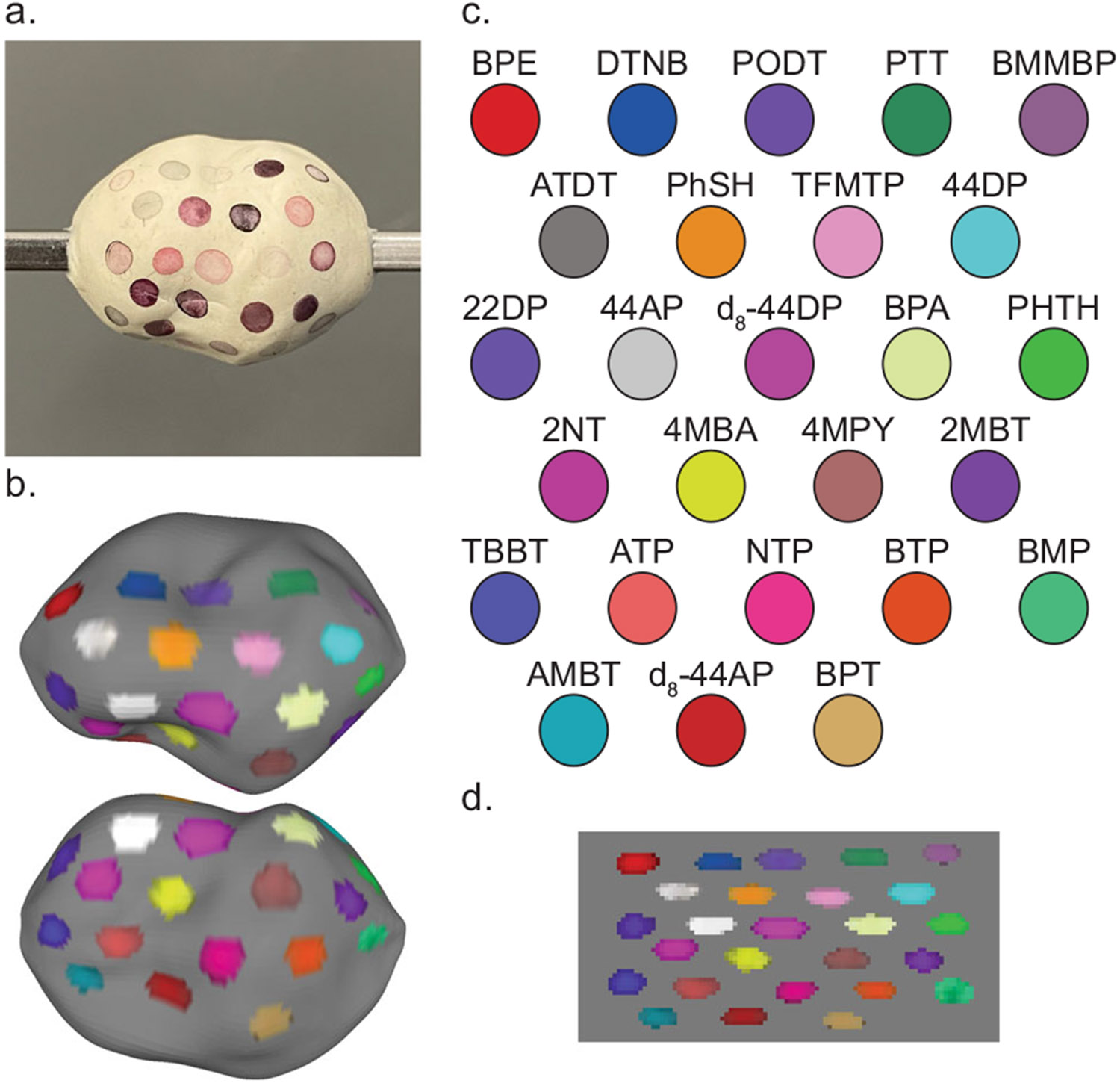
Topographic imaging with a large SERS NP library. A large array of SERS NP contrast agents can be fabricated and are compatible with topographic imaging. **a** Digital image of a phantom coated with 26 different SERS NP types. **b** Color-coded topographic image of the phantom. **c** Legend of Raman reporter abbreviated names and associated pseudocolors. **d** Unwrapped representation of the color-coded surface image.

The power of Raman imaging with SERS NP contrast agents comes from both the spectral distinctiveness of each molecule’s inelastic scattering profile and the vast possibilities for molecular structures which could be used as Raman reporters. We have previously demonstrated the multiplexing capabilities of Raman imaging with up to 26 SERS NP types and postulated how more can be discovered and tested^33,34,38^. We show in Fig. 5 that each SERS NP type can be distinctly recognized under topographic Raman imaging. Each of these SERS NPs can be detected with one incident wavelength and in a single Raman spectral imaging scan. The structures of encapsulated reporter molecules and their spectra are included in Fig. S3. Some reporters could be used in SERS NP batches to target cancerous biomarkers, some as orientation markings, and some could even be designed to target other important cell types such as immune cells. A multitude of biomarkers may be clinically useful to stain across a fresh lumpectomy specimen^42,54-56^.

### Multiplexed SERS specificity validation

Cells express and present a multitude of proteins on their membranes which can be stained to identify them and to judge whether they may be cancerous. Cancers are known often to be discernible from healthy tissue by their expression of inappropriate amounts or combinations of proteins capable of driving accelerated growth signaling^57,58^. Multiplexed molecular imaging aims to capture and depict the presence of many biological features simultaneously by the clever use of separable optical signals to convey multiple channels of information at once. A cocktail of biomarker protein-targeting SERS NPs could be formulated for intraoperative topographic imaging to target the kinds of proteins discovered by pathology of a preoperative biopsy or even to target a large panel of possible biomarkers to increase the likelihood of discovering positive margins with unexpected expression properties. It would be necessary for the SERS NP contrast agents bound to a lumpectomy specimen to be distinct enough to be correctly identified to discern foci with different expression patterns.

**Fig. 6:**
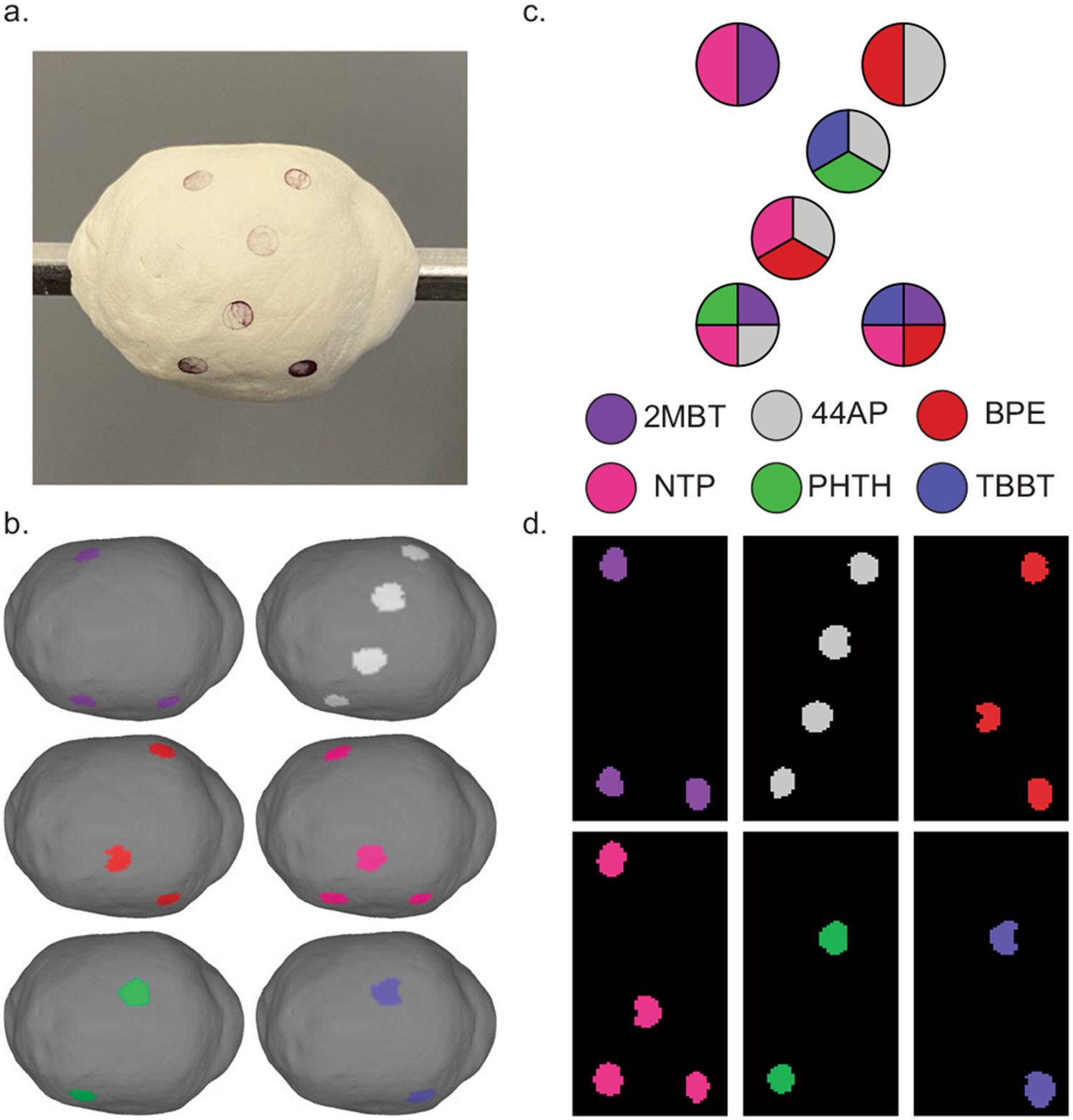
Multiplexed topography validation. SERS NPs can be mixed within the same pixels and still be correctly recognized and interpreted after quantitative spectral unmixing. **a** Digital photo of topography phantom coated with mixtures of SERS NPs. **b** Color-coded 3D topographic renderings of unmixed channels. **c** Key and legend of SERS NP mixture constituents. **d** Unwrapped representations of the color-coded surface images to reveal the location of specific SERS NP flavors.

A phantom of modeling material was coated with mixtures of SERS NPs, topographically imaged, and spectrally unmixed to reveal the contrast agents’ locations (Fig. 6). The key to each mixture’s SERS NPs was not revealed to the other members of the team prior to imaging and quantitative unmixing to ensure an unbiased assessment of the resulting channels. Plots of the SERS NP spectra included in each mixture is included in Fig. S4. Different SERS NP combinations and plexities were prepared to simulate conditions ranging from simple to more complex biomarker expression profiles. Six SERS NP types were chosen for this and later multiplexed topographic images due to the clinical relevance of 5 cancer biomarkers considered in the work of Wang et. al. which showed correct illustration of tumor locations and margins by staining the fresh surface of resected human tissue with biomarker-targeting SERS NPs^43-45^. In those stains, an additional isotype negative control SERS NP was conjugated with a nonspecific IgG antibody and included in the SERS NP staining cocktails to account for non-specific binding by taking unmixed signal ratios. We perform multiplexed validation with 6 SERS NPs to demonstrate the capability of this topographic imaging approach to handle detecting a relevant number of clinically and surgically useful biomarker protein targets.

### Multiplexed SERS Topography of Tissue

**Fig. 7:**
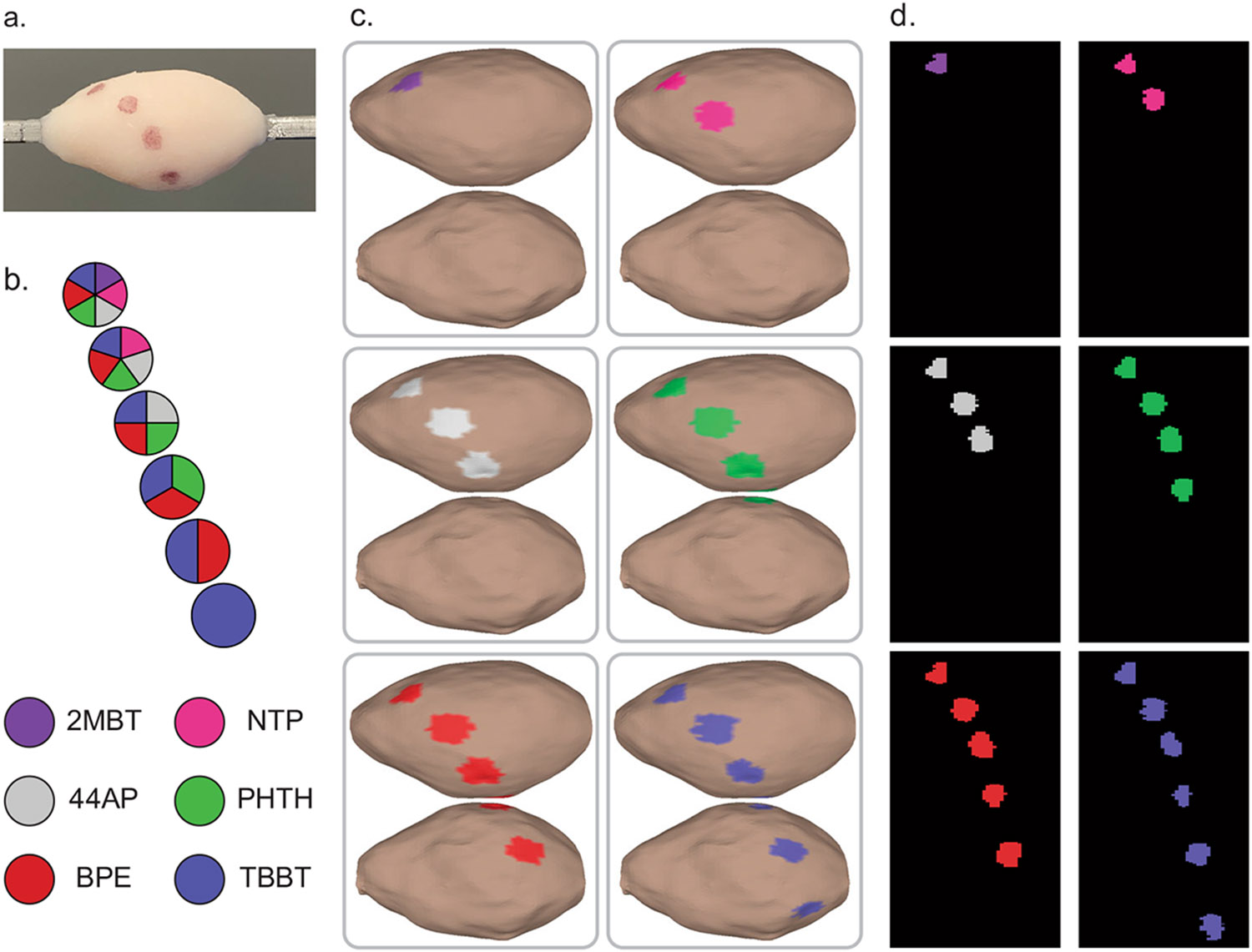
Topographic SERS NP multiplexing on tissue. Multiplexed signals are qualitatively discernible even when performing topography on tissue substrates. **a** Digital photo of a tissue specimen coated with mixtures of SERS NPs. **b** Key and legend of SERS NP mixture constituents. **c** Color-coded 3D topographic renderings of unmixed channels. **d** Unwrapped representations of the color-coded surface images.

We graduated to topographic imaging with SERS NPs on the surface of organic phantoms to demonstrate the imaging approach’s capability of autofocusing and topographically navigating real tissue. For this, we used porcine abdominal tissue as a human breast tissue phantom model. It is a commercially available tissue with a mixture of adipose, epithelial, and muscle content reasonably analogous to human breast tissue. Mixtures of SERS NPs were applied to the phantom surface and were correctly located upon Raman imaging with our rotational accessory device and subsequent spectral unmixing and interpretation (Fig. 7). Plots of the SERS NP spectra included in each mixture are included in Fig. S5. A clip of executing tissue phantom Raman topographic imaging with SERS NPs is included in Supplemental Video 3.

### Quantitative SERS topography of tissue

**Fig. 8:**
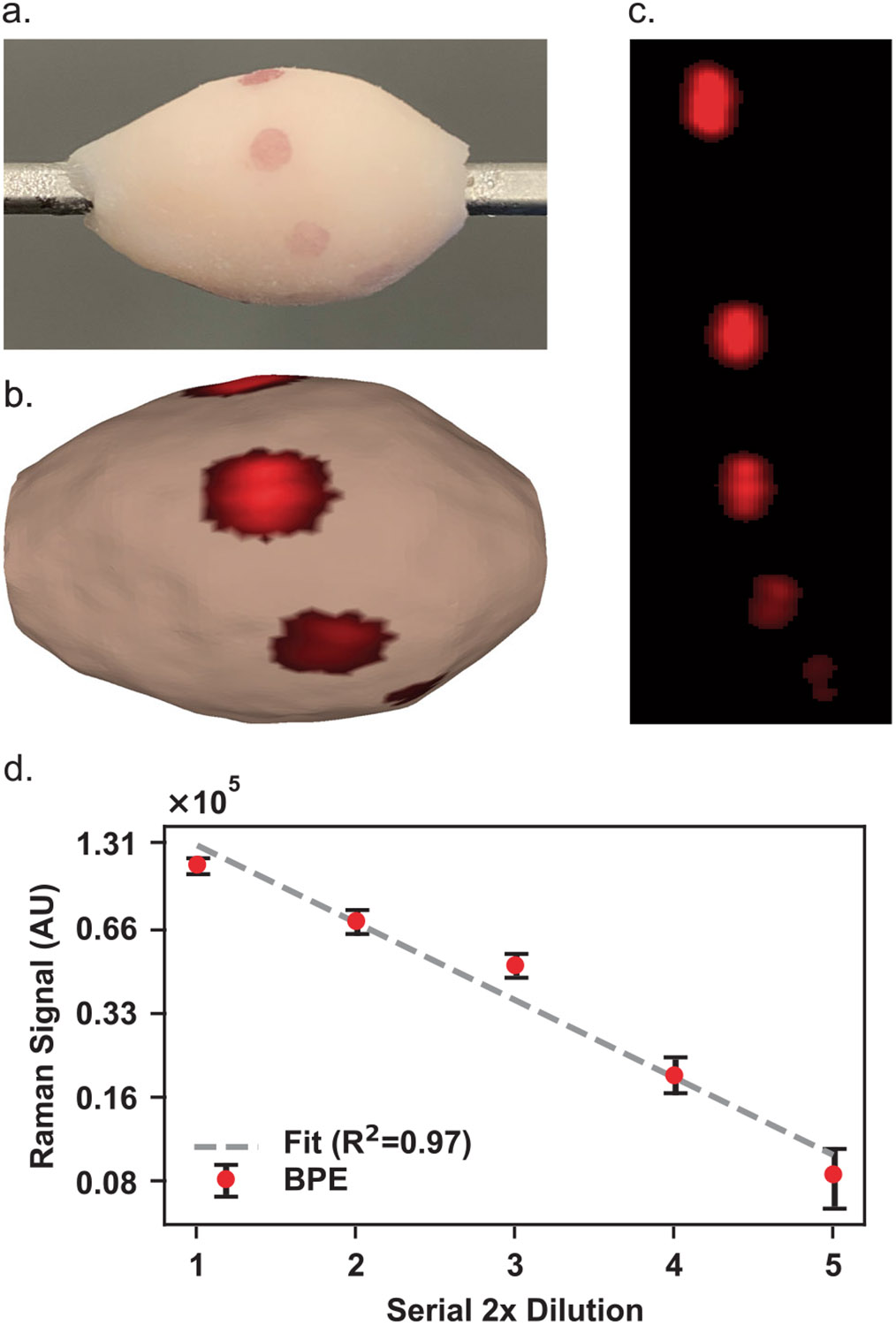
Quantitative topographic SERS NP imaging on tissue. Linear signal trend observed over serial dilutions of a SERS NP solution. **a** Digital photo of a tissue specimen coated with mixtures of SERS NPs. **b** Color-coded 3D topographic rendering of unmixed channel. Image has been log_2_-transformed to visualize SERS NP channel brightness gradient more clearly. **c** Unwrapped representations of the color-coded surface images. **d** Quantitation results based on the 1200 cm^−1^ BPE band plotted on log_2_-log_2_ axes with linearity R^2^ coefficient of determination of 0.97. Error bars represent standard error of the mean of region of interest (ROI) grayscale values.

SERS NP signals indeed attenuate proportionately with decreased local abundance. Quantitative behavior of an imaging system is often a desirable feature, even when the conclusions a clinician will draw from the image are qualitative or binary in nature. We show that topographic imaging of a sequence of a 2 × serially diluted SERS NP solution registers a highly linear Raman signal attenuation (Fig. 8). Though we recognize precise biomarker sensitivity may not be as significant of a factor as biomarker specificity, quantitative capabilities would be necessary to enable ratiometric biomarker binding evaluation and for non-specific binding thresholds to be set to ease image interpretation. Drops of diluted SERS NP solutions were placed at varying locations across the surface, and the Raman signals of the SERS NPs diminish linearly as expected. The BPE-labeled SERS NP concentrations applied in 3 μl aliquots to this tissue phantom are included in Table S1.

## DISCUSSION

We aim to address the clinical need for improved intraoperative tumor resection margin profiling of freshly excised tissue. Surgical treatment of breast cancer by tissue-conserving local lumpectomy suffers from poor visual tumor margin indicators during resection and current intraoperative imaging only helps the surgeon find the tumor, not check that the whole tumor and sufficient surrounding tissue has been removed. Breast cancer lumpectomy presently lacks methods to intraoperatively characterize margin status because its adipose content makes it incompatible with rapid cryosection-based histology. As breast cancer often presents with abnormal protein expression patterns, it is an ideal candidate for molecular imaging methods. Raman spectral imaging with SERS NP contrast agents is a powerful multiplexed imaging method which can be leveraged for molecular imaging applications by decorating the contrast agents with biomarker-targeting ligands to preferentially bind to known biomarkers of interest overexpressed on cancer cells. With an integrated sample-rotating accessory device, a commercial Raman imaging system can be extended to perform topographic imaging across the entire surface of an intact fresh tissue specimen. We found that the multiplexing and quantitative spectral properties of the SERS NPs fully translate to this new imaging method.

We show the capability of topographic Raman spectral imaging to accurately capture the size, shape, and texture of object surfaces while also spectrally unmixing and quantitating the presence of a large array of SERS NP contrast agents. We successfully located each of the 26 SERS NP flavors curated in our extensive library, specifically unmixed subsets of simultaneously multiplexed SERS NPs on real tissue phantoms, and linearly quantitated serially diluted SERS NPs with R^2^ coefficient of determination of 0.97. In practice, each SERS NP type could be deployed in a staining cocktail to highlight different biomarkers and therefore different cell populations which could be significant to a surgeon’s clinical evaluation to revise resection margins. Topographic imaging with multiplexing and quantitation was successful on actual tissue phantoms, demonstrating promise for future efforts to translate this imaging approach toward the operating room. Quantitation capacity is important and advantageous in a molecular imaging modality because it enables contrast agent abundances and therefore biological expression levels to be accurately analyzed and objectively compared. We show here and in previous work that protein expression levels by cells can be robustly assessed with quantitative SERS NP imaging^34,59^. With SERS NP flavors each designed with antibodies to target a multitude of biomarkers and a flavor conjugated to an isotype control antibody, various levels of relevant proteins could be evaluated and specifically localized across a tissue specimen’s surface. In addition, numerous cell types in the tissue microenvironment, including tumor cells, immune cells, stromal cells, and epithelial cells, could all be readily differentiated and contribute to the clinical assessment of a tissue specimen. This imaging can be further developed and validated by acquiring human breast cancer tissue, performing SERS NP biomarker stains, and corelating positive margins with traditional FFPE pathology. The topographic imaging accessory device may undergo further refinement to enable affixing specimens without the need for a piercing rod. As standard of care postoperative pathology may be interested in evaluating the unaltered expression and morphology of a specimen’s interior, an alternative strategy could be devised to gently and nondestructively secure specimens to the rotation device. We have focused here on applying topographic SERS Raman imaging to address the needs of breast cancer lumpectomy, but the principles may be generalized to apply to the resection of other solid tumors that suffer from poor margin delineation such as head and neck cancers with identifiable protein expression patterns^25,26,60-63^.

Some biomarkers, which have been mentioned here, and researched for their clinical utility in locating and characterizing breast cancer include EGFR, HER2, ER, CD24, CD44, and CD47. These markers are extensively studied in breast cancer because they carry biological significance. EGFR is overexpressed in several tumors and is associated with tumor epithelial-mesenchymal transition (EMT), migration, and invasion^64^. Triple negative breast cancers (TNBCs), which lack overexpression of progesterone receptor (PR), HER2, and ER, may still overexpress EGFR in most cases^64^. HER2 is a receptor tyrosine kinase in the same family as EGFR upstream of cellular growth signaling pathways and is overexpressed in up to 25% of breast cancer cases^65-69^. ER overexpression is found in approximately 65% of breast cancer cases, is correlated with favorable response to endocrine therapy, and can be detected on the cell membrane^70-76^. Cluster of differentiation (CD) protein markers signify cells’ immunophenotype by their role or relationship to the immune system and can also be targets to localize breast cancer. CD24 and CD44 expression on breast cancer cells have been shown to correlate with disease aggressiveness and risk of metastasis^77-79^. CD47 expression has also been shown to be overexpressed in several breast cancer cell lines and is associated with overall increased cancer cell survival^42^. CD47 is involved in evading the immune system and in tumor relapse^80^. Since TNBC patients account for about 20% of breast cancer patients, investigators are also studying other important protein biomarkers that have shown promise as targets for breast cancer such as folate receptor (FOLR) or programmed death-ligand 1 (PD-L1)^81,82^. TNBCs have also been characterized by increased CD8+, CD4+, and FOXP3+ lymphocytic infiltration^83^. Biomarkers such as these could also be used to detect margins and suggest treatment routes for uncommon breast cancer cases like TNBC.

Raman spectral imaging with SERS NP contrast agents is a highly promising tool for molecular imaging. We have validated the capabilities of these spectral signals to be distinguishable and quantifiable, even in complex 3D topographic surface imaging scenarios with varying illumination angles. It is a powerful and flexible imaging technology which can be adapted to demanding clinical settings in need of molecular imaging solutions. Spectral distinctiveness of SERS NP contrast agents is guaranteed by the quantum properties of Raman scattering, and optical sensitivity enhancement is provided by the nanometallic core. Many possible cell types along a resection margin could also be readily stained and interrogated using the vast multiplexing capabilities of SERS NPs, opening the possibility to evolve margin resection protocols and improve patient outcomes.

## Supplementary Material

SI

SI Video 3

SI Video 2

SI Video 1

## Data Availability

The data that support the findings of this study are available from the corresponding author upon reasonable request.
