# Supplementary material for: A Raman topography imaging method toward assisting surgical tumor resection
Source: Npj Imaging. 2024 Feb 19;2:2. doi: 10.1038/s44303-024-00006-6 (PMC11884652; doi:10.1038/s44303-024-00006-6)
Supplement: Supplementary file 1 — Supplementary Information [file 44303_2024_6_MOESM1_ESM.pdf]

Supplementary Information

A Raman Topography Imaging Method Toward Assisting Surgical Tumor Resection

Alexander Czaja[1,2], Alice J. Jiang[1,2], Matt Zacchary Blanco[1,2], Olga E. Eremina[1,2], Cristina Zavaleta[1,2]

1 Department of Biomedical Engineering, University of Southern California, 3650 McClintock Ave, Los Angeles, CA 90089, United States
2 Michelson Center for Convergent Bioscience, University of Southern California, 1002 Childs Way, Los Angeles, CA 90089, United States

Corresponding Author
* czavalet@usc.edu

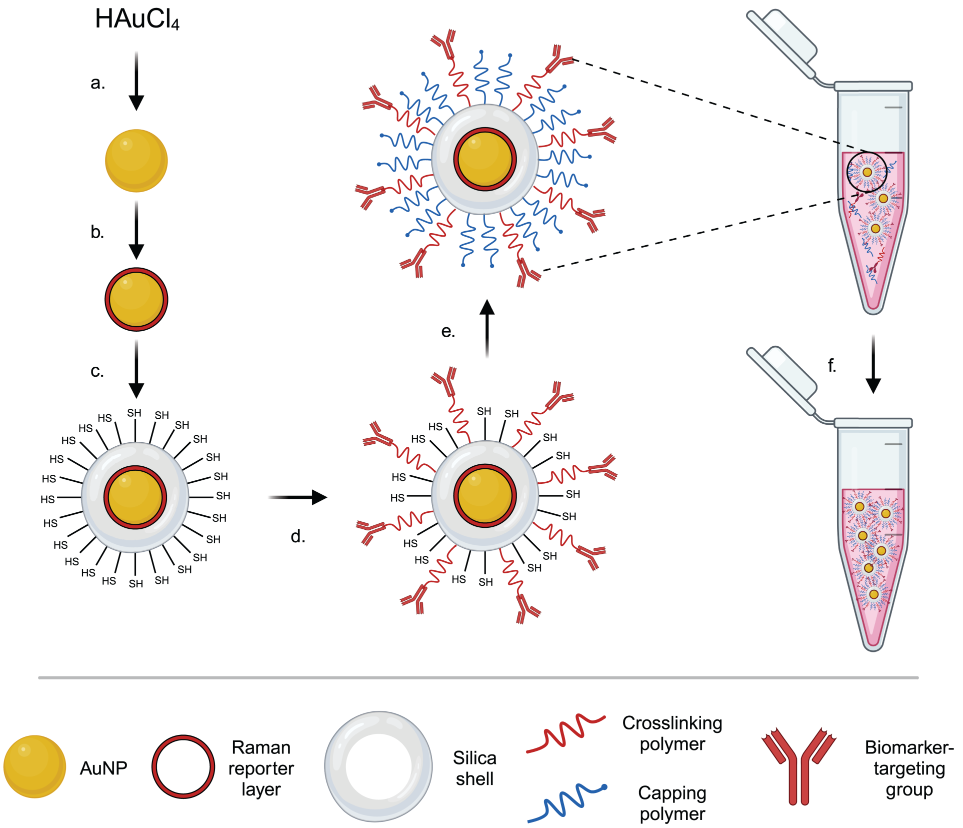

Figure S1 – SERS NP fabrication: a. The gold nanoparticle (AuNP) core is formed, b. a solution containing a Raman reporter is added and coats the AuNPs, c. a protective thiolated silica shell is formed, d. biomarker-targeting antibodies are conjugaed to the NPs with linking groups, e. free thios are capped, f. free reagents are washed by centrifugation.

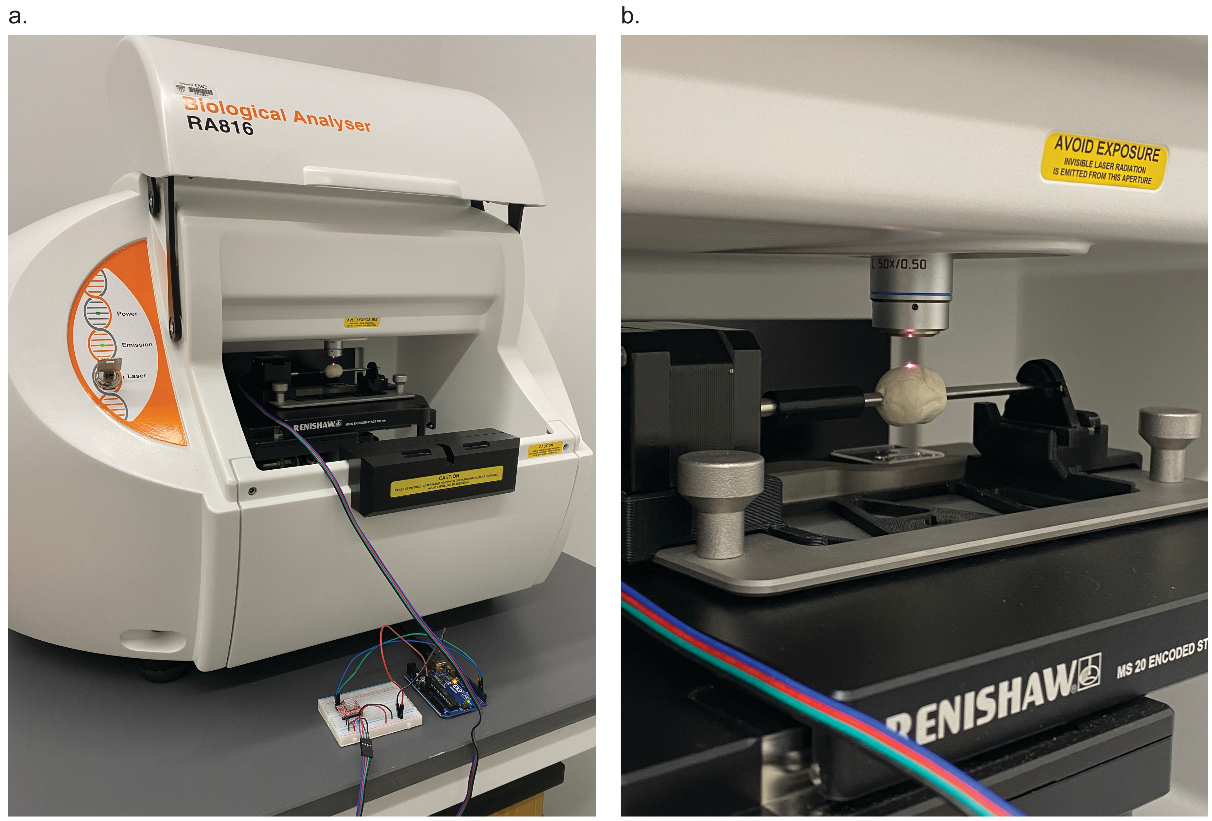

Figure S2 – Commercial benchtop Raman imaging system with topography accessory: The Renishaw RA816 Biological Analyser is a user-friendly and extensible Raman spectral imaging system. It is fully contained and small enough to fit on a standard laboratory workbench. Our topographic imaging accessory device is designed to accommodate the space constraints inside the Raman imaging system and rotate samples on-demand during Raman imaging. Topographic imaging is achieved by rotating samples on the accessory's rod incrementally while the stage steps horizontally. This method effectively samples pixels from a sample's left to right side along its whole surface in a spiral fashion. a. RA816 system with topographic imaging accessory. b. Device and a sample on the stage being topographically imaged with the RA816's autofocus system keeping the sample surface in focus.

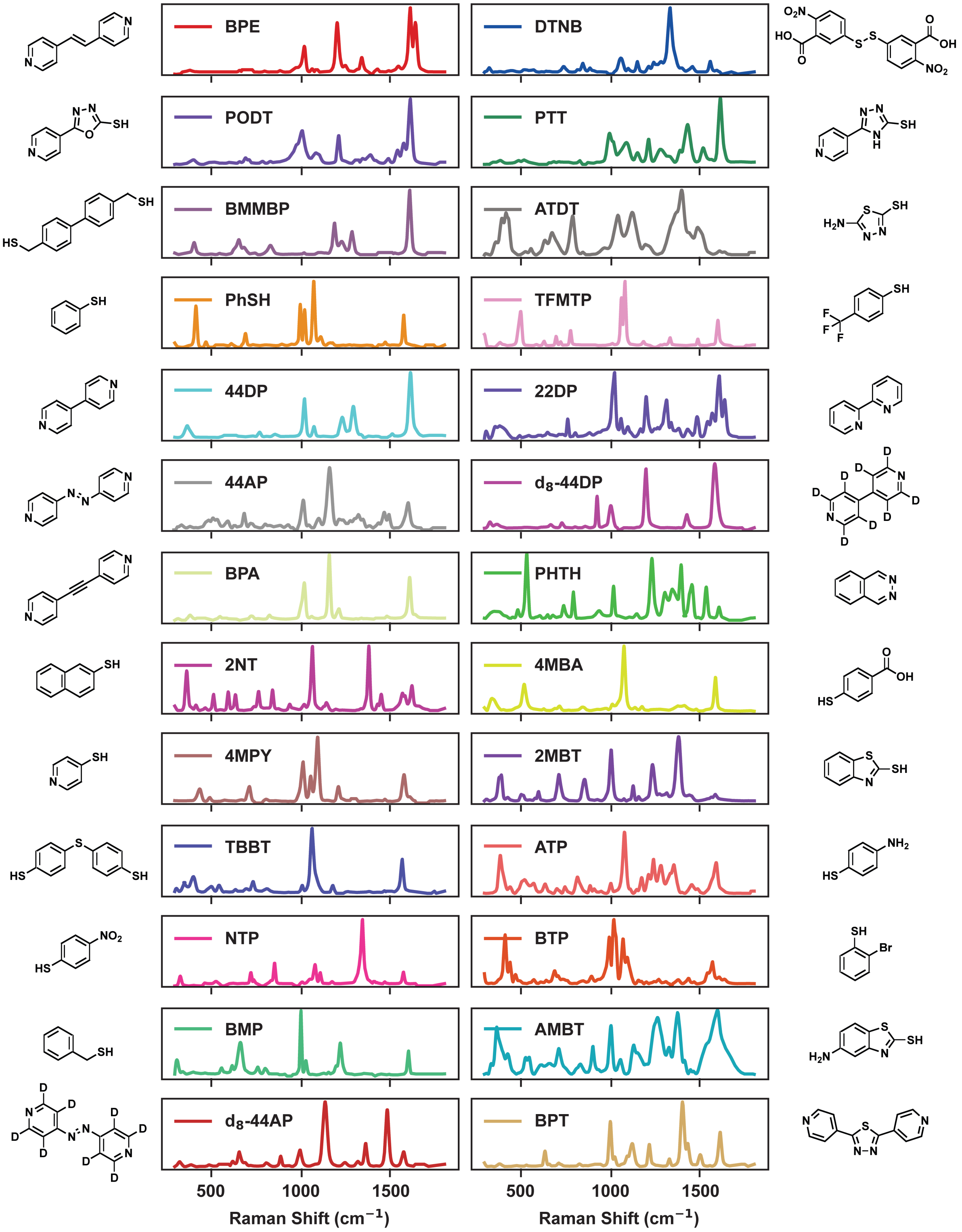

Figure S3 – Library of SERS NP reporters: Structures of Raman reporters along with plots of their respective SERS spectra. The atomic makeup, bonds among the atoms, and structural and electron system symmetries all play roles in their respective, distinct inelastic scattering profiles.

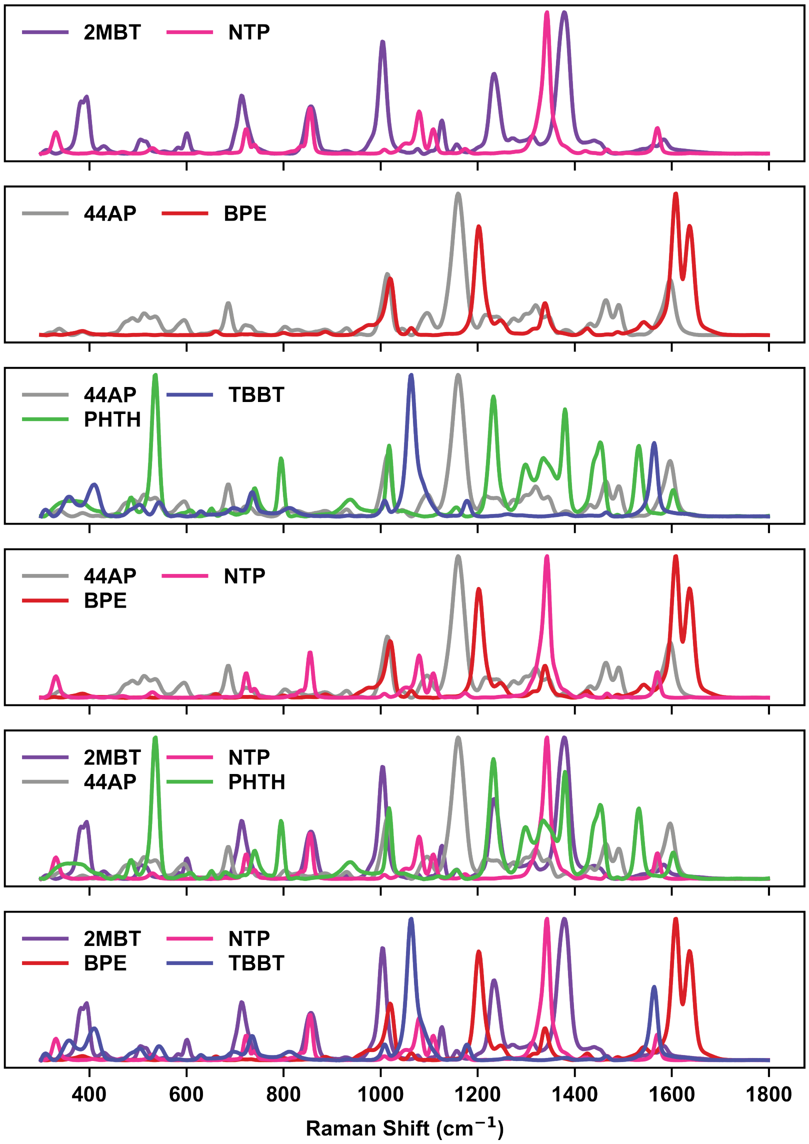

Figure S4 – Multiplexed topography validation mixtures: Mixtures of SERS NP contrast agents deposited onto a modelling material phantom ranging from 2-plex to 4-plex with randomly chosen agents. The mixture constituents were all able to be readily identified by spectral unmixing in the rendered topographic image results.

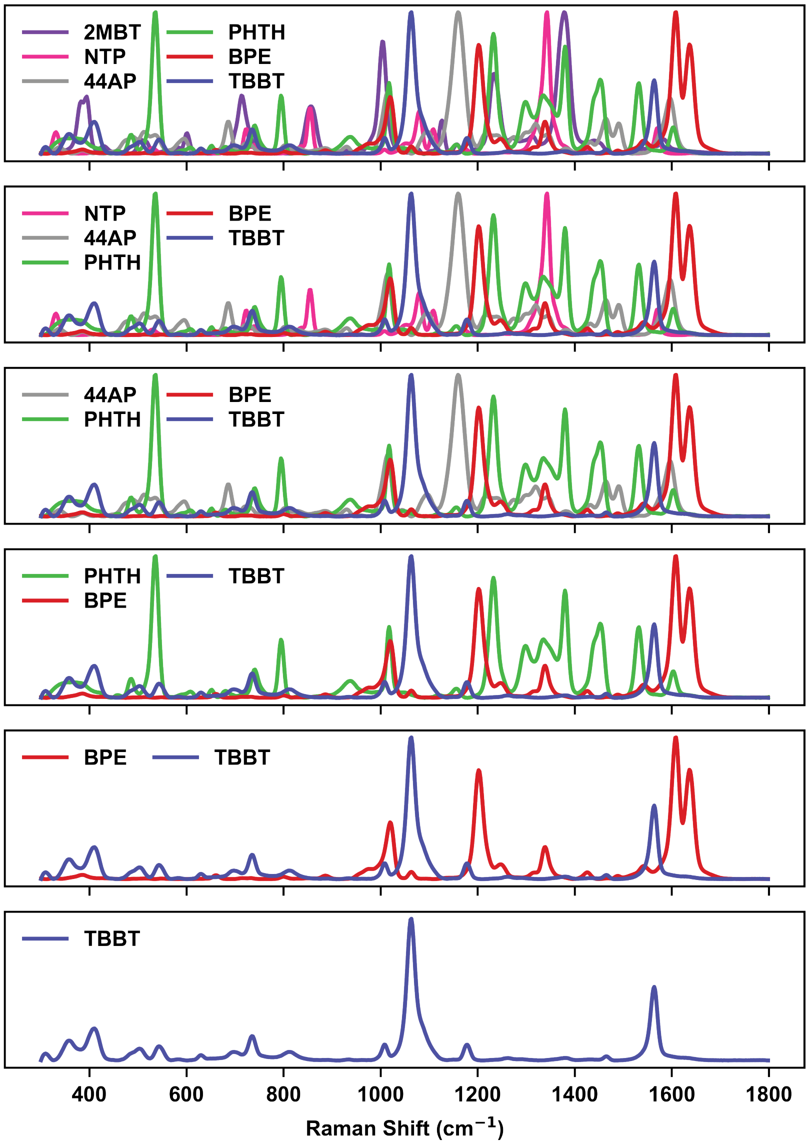

Figure S5 – Multiplexed SERS NP multiplexing on tissue mixtures: Mixtures of SERS NP contrast agents deposited onto a porcine abdominal tissue phantom ranging from 1-plex to 6-plex. The mixture constituents were all able to be readily identified by spectral unmixing in the rendered topographic image results.

Serial Dilution	Dilution	Concentration (pM)
1	2×	324
2	4×	162
3	8×	81
4	16×	40
5	32×	20

Table S1 – NP Concentrations deposited for quantitative tissue topography: Concentrations were measured by measuring the UV-Vis absorbance at 520 nm of the BPE SERS NP stock solution used for preparing the serial dilution. 3 µl aliquots of each dilution were transferred onto the porcine abdominal tissue phantom for quantitative topographic imaging.
